# Relationship between mucosa-associated gut microbiota and human diseases

**DOI:** 10.1042/BST20201201

**Published:** 2022-10-10

**Authors:** Nathalie Juge

**Affiliations:** Quadram Institute Bioscience, Gut Microbes and Health Institute Strategic Programme, Norwich NR4 7UQ, U.K.

**Keywords:** gut microbiota, mucosa-associated microbiota, mucus

## Abstract

The mucus layer covering the gastrointestinal (GI) tract plays a critical role in maintaining gut homeostasis. In the colon, the inner mucus layer ensures commensal microbes are kept at a safe distance from the epithelium while mucin glycans in the outer mucus layer provide microbes with nutrients and binding sites. Microbes residing in the mucus form part of the so-called ‘mucosa-associated microbiota’ (MAM), a microbial community which, due to its close proximity to the epithelium, has a profound impact on immune and metabolic health by directly impacting gut barrier function and the immune system. Alterations in GI microbial communities have been linked to human diseases. Although most of this knowledge is based on analysis of the faecal microbiota, a growing number of studies show that the MAM signature differs from faecal or luminal microbiota and has the potential to be used to distinguish between diseased and healthy status in well-studied conditions such as IBD, IBS and CRC. However, our knowledge about spatial microbial alterations in pathogenesis remains severely hampered by issues surrounding access to microbial communities in the human gut. In this review, we provide state-of-the-art information on how to access MAM in humans, the composition of MAM, and how changes in MAM relate to changes in human health and disease. A better understanding of interactions occurring at the mucosal surface is essential to advance our understanding of diseases affecting the GI tract and beyond.

## Introduction

**Figure 1.**
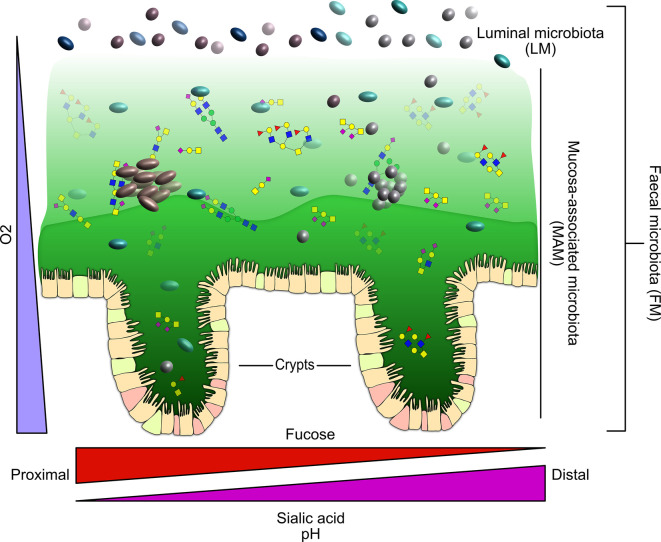
Schematic representation of the colonic mucosal interface. The epithelium surface is covered by a mucus bilayer with the outer mucus layer facing the lumen being a habitat for the microbiota while the inner layer provides a protection from microbial invasion and luminal content. The mucin glycans, O_2_ and pH gradients all contribute to shaping the mucosa-associated microbiota (MAM).

The community of microbes, including bacteria, archaea, viruses and fungi, that inhabit the gastrointestinal (GI) tract, collectively referred to as the gut microbiota, has a profound impact on human health [1]. The spatial organisation of the gut microbiota along and across the GI tract influences host–microbe and microbe–microbe interactions [2–4]. In the GI tract, the epithelium surface is covered with mucus, a gel made of water, electrolytes, lipids and various proteins [5]. The colon has relatively well-defined and continuous mucus layers, in contrast with the small intestine, where the mucus is patchy and penetrable [6–7]. In the colon, where the density of bacteria is higher than elsewhere in the GI tract, mucus protects the epithelium from microbial damage and luminal compounds, but also plays a major biological role by harbouring a microbial community called the ‘mucosa-associated microbiota’ (MAM). This is enabled by the bilayer organisation of the colonic mucus into a loose outer layer that provides a niche for microbes adapted to this environment and a stratified inner layer that restricts bacterial access to the epithelium [6,8] (Figure 1). However, this description needs to be nuanced to reflect local and host species-related differences along the length of the colon. In mice, while the inner mucus layer of the distal colon was shown not to be penetrable by beads of comparable size to bacteria, the inner layer of the proximal colon was partly penetrable, as in the small intestine [7]. The goblet cells of the luminal surface epithelium mediate fast turnover (1 h) of the inner mucus layer in the distal colon as shown using *in vivo* metabolic labelling in mice [9]. While mucus is replaced at a rate of 240 and 100 μm/h in human and mouse explants, respectively [10], colonic mucus organisation and thickness (inner mucus thickness estimates ≈50 µm in mice and ≈200 µm in humans) is also intimately dependent on colonisation by the gut microbiota [11–13] and dietary fibre intake [14] with a gradient of increasing thickness along the length of the colon [6]. This spatial organisation is disturbed in the absence of dietary fibre, resulting in local changes along the colon, impairment of the mucus layer and increased susceptibility to infection [15–19]. In addition, recent studies reported that the organisation of the colonic mucus was shaped by both colonic content and location within the colon [20]; that bacteria were detected in the inner mucus layer [21]; and that mucus-embedded microbiota were present in faecal samples [22].

MAM contributes to gut barrier function by stimulating the production of mucus [12,23] and antimicrobial compounds against pathogenic bacteria [24]; by competing with pathogen species for space and nutrients [25–26]; and by producing metabolites directly implicated in communication between microbes and the host [17,27–28]. MAM also plays a role in the maturation of the host immune system [29]. While the luminal microbiota (LM) responds primarily to changes in diet, MAM may be more directly influenced by host-related factors [19,30]. Importantly, the ability to use host mucin glycans as a carbon source provides bacteria with a sustainable and consistent nutrient supply and a competitive advantage during colonisation of the mucus layer [30–32]. Other niche-specific factors contributing to spatial variation in microbial distribution include the pH and O_2_ gradients along and across the GI tract [33] (Figure 1). As a result of fibre fermentation and the production of short-chain fatty acids (SCFAs), there is a luminal (longitudinal) pH gradient that results in a slightly acidic pH in the proximal colon which then increases again distally towards the rectum [34–36]. However, the mucus layer also influences the pH transversally by capturing hydrogen and bicarbonate ions and protecting the epithelium from luminal pH changes. The O_2_ concentration changes along the two axes of the gut with a decreased transversal gradient from the highly vascularised intestinal mucosa to the gut lumen [37] and a decreased longitudinal O_2_ gradient from the duodenum towards the colon due to changes in epithelial cells and microbial metabolism [38]. These changes in mucus, pH and O_2_ concentration create local environments that influence the structure and function of the MAM along the GI tract. To date, most of the knowledge about mechanisms of microbiota adaptation to the mucus niche is based on pre-clinical models and only a few studies have investigated the structure and function of the MAM in humans.

## Mucin glycosylation is an important element in the regulation of the mucosa-associated microbiota

The biological and physico-chemical properties of mucus are largely attributable to mucins, the major structural components. Mucins are membrane-bound, gel-forming or secreted, and vary along the GI tract [32]. Secreted gel-forming MUC2 in the human large intestine (Muc2 in rodents) is produced by specialised goblet cells. A close symbiotic relationship exists between the gut microbes inhabiting the mucus niche and the mucin glycans that make up ∼80% of the molecular mass of mucins.

Mucin *O*-glycosylation is characterised by a high degree of structural diversity. Mucin *O*-glycosylation is initiated by a large family of polypeptide GalNAc transferases that add *N*-acetylgalactosamine (GalNAc) to Ser and Thr residues of the mucin backbone, resulting in the formation of the Tn antigen, which represents the substrate for further additions of sugars by glycosyltransferases to form mucin core structures. There are eight mucin core structures in humans; cores 1–4 are the most common structures in intestinal mucins [32,39]. These core structures are further elongated by the addition of galactose, GalNAc and/or *N*-acetylglucosamine (GlcNAc) residues leading to linear or branched chains of up to 20 residues [40]. These glycan chains are often terminated with fucosylated, sialylated or sulfated epitopes [32].

Mucin glycosylation profiles show region specificity along the GI tract [40–43]. The terminal epitopes show considerable variation with a decreasing gradient of fucose and ABH blood group expression and an increasing gradient of blood group Sd(a)/Cad-related epitopes and sialic acid from the ileum to the colon in humans [42–43] and reverse gradients in mice where the small intestine is dominated by sialylated structures and the colon with those terminating in fucose [41]. The gut microbiota induces Muc2 production from proximal colon goblet cells and in turn, Muc2 *O*-glycans modulate the structure and function of the microbiota as well as transcription in the colon mucosa, demonstrating the critical role of mucin glycosylation in host–microbiota symbiosis [22].

At the mechanistic level, microbes adapted to the mucus niche are equipped with a range of adhesins [44] and a repertoire of carbohydrate-active enzymes (CAZymes) of diverse substrate/linkage specificity allowing them to break down mucin glycan chains [45–48] for direct use or through cross-feeding, as extensively reviewed [32,48–51]. Studies in mice confirmed that bacterial species display specific genomic repertoires that allow them to persist in the outer mucus layer better than the same species in the intestinal lumen [52] , underscoring the importance of CAzymes in this niche adaptation [15,53]. It should also be noted that no mucus-specific taxa were detected, and it was proposed that the mixing of mucus-associated bacteria and lumen bacteria is likely to occur in the colon perhaps through the renewal of mucus, although favouring the expansion of species better adapted to the mucosal compartment niche such as *Akkermansia muciniphilia* [17,54–55].

## How to profile mucosa-associated microbiota in humans?

Faecal samples are frequently used as a non-invasive proxy for studies of the gut microbiota because they are easy to collect and provide sufficient material for downstream analyses. However, stools do not account for regional changes in microbial communities occurring along and across the GI tract. Growing evidence suggests that the faecal microbiota (FM) differs from the intestinal MAM and is most similar to the LM from the descending colon, as described in the next section. In addition, several studies suggest that the tightly adherent and loosely associated mucosal communities of the gut contain functionally distinct bacterial communities, and that some microbes are found in intestinal crypts. However, variability in the anatomical sites of collection, methods of sampling, sample size, methods of sequencing and bioinformatics pipelines has often led to conflicting results in terms of MAM composition, highlighting the need for more standardised approaches to sampling and profiling these microbial communities.

Currently, the main means of characterising the intestinal MAM are endoscopic biopsies, but these present a series of drawbacks as recently reviewed [56–57]. In theory, colonic biopsies should contain microbial communities from both the outer and inner mucus layers. However, in most human studies, intestinal biopsies are collected after colonic lavage which may affect the integrity and abundance of the mucus and associated microbiota as demonstrated in a study using biopsies from individuals undergoing un-prepped flexible sigmoidoscopy or standard polyethylene glycol-based bowel cleansing preparation [58]. In addition, biopsy collection is an invasive procedure compared with stool sampling and represents a discrete area of the mucosa that may not be representative of the overall MAM. At a more technical level, biopsies contain a small biomass of microbiota and a higher proportion of host DNA which provides technological challenges for accurate profiling of MAM from the host or exogenous microbial contaminants. Finally, the small size of intestinal biopsies and the limited number that can be collected from the same site, reduce replication and the analytical power that can be achieved. Laser capture microdissection (LCM) of biopsies overcomes some of the drawbacks by enabling a more accurate capture of specimens in the crypt-associated mucus and mucus layers [21,59–60].

Rectal swabs and gentle scraping of the mucosa with brushes may provide less invasive sampling methods that also reduce contamination by human cells in metagenomics analysis [61]. However, it has been reported that bacterial communities from unprepared rectal swabs were very similar to those in stools [62–63], therefore limiting the application of this approach to MAM studies. Recently, samples taken from the ileum end, ascending and sigmoid colon by brushing during colonic endoscopy were used for MAM profiling and compared with the FM [64]. Despite the limitations of the study in terms of sample size and use of bowel preparation for colonoscopy, the results suggested that brushing could be used to profile more adherent MAM [64] while intestinal ‘lavage’ (i.e. fluid remaining in the bowel after bowel preparation) was proposed as a proxy for profiling microbial communities remaining in the lumen after bowel preparation and in the loose mucus layer [65].

An alternative procedure for collecting MAM involves flushing the mucosal surface with sterile water and aspirating the resulting mucus suspension. This technique allows for the mucosal–luminal interface (MLI) to be sampled by washing off and collecting the loose mucus layer on the surface of the intestinal wall [56,66]. Mucosal lavage is from a defined (∼1 cm^2^) area of the mucosal surface, and therefore captures the local microbial community in interaction with the microenvironment thereby mimicking the regional specificity of biopsies while providing sufficient biomass for in-depth analysis (40–80 ml of aspirate vs 5 ml for colonic lavages). Another advantage is that processing and storage of MLI samples has minimal impact on the microbial composition as assessed by 16S rRNA amplicon sequencing [56]. Although the collection of MLI aspirates is still an invasive approach, it is less damaging to the epithelium than biopsies, especially for vulnerable patients. To avoid any interference with MAM analysis, Mottawea et al. [56] also recommended the exclusion of mucolytic reagents such as dithiothreitol or *N*-acetylcysteine that may remove bacteria associated with the colonic outer mucus layer.

In conclusion, several approaches are available to study gut microbes at the mucosa interface in humans and, depending on the sampling method, these can target adherent (biopsies, brushing) or more loosely attached (MLI, lavages) mucosal communities. A common limitation of these approaches is the need for patients to undergo an endoscopic procedure which explains the limited number of studies addressing MAM and the generally low sample size compared with studies investigating faecal microbiomes. There is also agreement across studies that bowel preparations prior to endoscopy affect the MAM, as and that the low biomass in biopsies can impede sequencing and downstream analyses. Although at an early stage of development, swallowable bacteria-sampling capsules (e.g. [67]) that sample along and across the GI tract may be a way forward to overcome issues related to the invasiveness of the procedures.

## Can we define a mucosa-associated microbiota in humans?

MAM is an undefined term covering heterogenous microbial communities that can be attached to the mucosa or loosely associated with mucus depending on the mode of sampling (as described above). Another limitation, which is also true but to a lesser extent for FM, is that knowledge about the MAM is limited to bacteria and very little is known about fungi and viruses in those samples. In addition, due to the invasiveness of sampling procedures, most knowledge about MAM in humans is derived from the analysis of samples from diseased patients who have required endoscopy as part of their treatment, e.g. patients with inflammatory bowel disease (IBD), irritable bowel syndrome (IBS) and colorectal cancer (CRC) (see next section).

Given the limited number and size of studies and the heterogeneity of analysed samples, it is not possible to define MAM composition in healthy individuals; what is clear is that the composition of the MAM differs from that of the LM and the FM. This was first reported two decades ago using molecular profiling approaches such as denaturing gradient gel electrophoresis (DGGE) analysis [68–70] or temporal temperature gradient gel electrophoresis (TTGE) [71] in biopsy samples. Spatial heterogeneity and co-occurrence patterns of mucosal microbiota along the length of the human GI tract was further demonstrated using 454-pyrosequencing of bacterial 16S rDNAs associated with biopsy from terminal ileum, ileocecal valve, ascending colon, transverse colon, descending colon, sigmoid colon and rectum [72,73]. More than 90% of healthy human gut bacterial species belong to four major phyla: Bacteroidetes, Firmicutes, Actinobacteria and Proteobacteria [74] but the relative abundances of the main phyla in MAM appears to be study-specific [74–76]. In a recent study limited to 23 participants, the most abundant bacterial genera in the colonic mucosa were found to be *Bacteroides, Faecalibacterium, Escherichia/Shigella, Sutterella, Akkermansia, Parabacteroides, Prevotella, Lachnoclostridium, Alistipes, Fusobacterium, Erysipelatoclostridium* and several *Lachnospiraceae* family members. The bacterial community composition was homogeneous across the large intestine while inter-individual variability was greatest between the cecum and the rectum. Despite the small number size of participants (n=13), significant differences in biodiversity and the taxonomic structure were observed between rectal and FM [73]. In a recent study using laser-dissected tissue from colonic biopsies (mostly descending colon), Firmicutes and Proteobacteria were the most abundant phyla while Actinobacteria and Bacteroidetes were only detected at low levels [77]. In another study, MAM composition was determined from mucosal biopsies from nine different sites in individuals undergoing antegrade and subsequent retrograde double-balloon enteroscopy. MAM changed along the GI tract with larger bacterial load, diversity and abundance of Firmicutes and Bacteroidetes in the lower GI tract than the upper GI tract, which was predominated by Proteobacteria and Firmicutes [78]. These inter-study discrepancies could be due to differences in the site and method of sampling as a recent study comparing microbial communities from aspirates, biopsies or stools showed a gradual change in the Bacteroidetes and Firmicutes ratio from biopsies to aspirates to stools [56]. It is also worth noting that most of these studies relied on 16S amplicon sequencing data and so some data variation could be attributed to sample storage, DNA extraction methods, region of the 16S rRNA gene amplified, library preparation and data analysis methods. The use of shotgun metagenomic sequencing instead of 16S amplicon to investigate MAM in intestinal biopsies is hampered by high-level contamination with host genome content resulting in insufficient sequencing depth [79].

In addition, despite the accepted notion that MAM is confined to the outer mucus layer, the use of LCM in combination with metagenomics has revealed that some commensal bacteria can be associated with the inner mucus layer and colonic crypts in healthy individuals. These studies revealed that the microbial community of colonic crypts was dominated principally by *Acinetobacter* spp, usually rich in Proteobacteria [80] and that the inner mucus layer was characterised by communities comprising 20–60% Proteobacteria, with fewer Bacteroidetes and a higher level of species (α)-diversity than found in faecal samples [21].

To date, knowledge about the MAM is largely limited to its composition. One study based on mucosal lavage samples from different intestinal locations showed significant differences in the mucosal metaproteome between the proximal and distal colon, implying distinct functionality within specific intestinal niches [66].

## How do changes in mucosa-associated microbiota relate to human diseases?

Attention is increasingly focussing on MAM of the GI tract in patients with a variety of intestinal diseases such as IBS, IBD and CRC as described below. However, evaluating the relationship between MAM and human diseases is confounded by variations in sampling method (as highlighted above), profiling methods (FISH, qPCR, metagenomics), statistical analyses, the size of the studies, as well as the severity of disease, sample type and origin in the GI tract.

### Inflammatory bowel disease

IBD includes Crohn's disease (CD) and ulcerative colitis (UC). Substantial data from experimental models and clinical studies suggest that the gut microbiota plays an important role in the pathogenesis of IBD and a growing number of studies highlight the relevance of spatial composition in the microbial changes associated with IBD.

Early studies focused on the analysis of mucosal tissue from IBD patients and showed an increase in the abundance of mucosa-associated bacteria [81]. For example, compared with healthy mucosa, *Ruminococcus torques* and *Ruminococcus gnavus* were more abundant in CD and UC mucosa while the main mucolytic bacterium *Akkermansia muciniphila* was significantly less abundant in both CD and UC mucosa [82]. Low abundances of *Faecalibacterium prausnitzii* [83–84], *Clostridium leptum* and *Prevotella nigrescens* subgroups were observed in the small intestine of CD patients [85]; low counts of *F. prausnitzii* were also found in the FM of patients with active IBD [86]. High prevalence of aggregative, adherent *Escherichia coli* strains has been reported in the ileum of CD patients and in the rectum and sigmoid of both UC and CD patients [87–89]. These differences in microbial composition are influenced by the site and stage of the disease with active UC patients having significantly lower microbial diversity throughout the GI tract [90]. Sequencing analysis of biopsy and faecal samples from IBD patients showed significant reductions in the proportion of several butyrate-producing bacteria (e.g. *Roseburia*, *Coprococcus* and *Ruminococcus* species) in MAM of UC patients compared with healthy individuals, in line with earlier work [91] whereas *Escherichia–Shigella* and *Enterococcus* pathogens were most prevalent in patients with IBD [92]. Analysis of intestinal biopsies by fluorescence *in situ* hybridisation (FISH) identified *Bacteroides fragilis* biofilms as the main feature of IBD [93]. More recently, the use of both 16S rRNA transcript and gene amplicon sequencing revealed that the abundant microbiota members of the inflamed tissue in UC patients were not the most active [94].

In the past decade, samples other than biopsies have been used to investigate MAM changes in IBD patients. For example, 16S rRNA sequencing of endoscopic lavages from different intestinal regions in 64 subjects (32 controls, 16 CD and 16 UC patients in clinical remission) showed a reduction in phylogenetic diversity and shifts in microbial composition in CD and UC patients; with distinct microbial metabolic functionality classifying the IBD status of individual patients during disease quiescence [95]. More recently, the colonic MAM profiles of patients with IBD were determined using mucus samples taken by gentle brushing of mucosal surfaces using endoscopic cytology equipment from the ileum, caecum and sigmoid colon of 43 patients with UC, 26 with CD and 14 non-IBD controls [96]. Although no significant differences in microbial community structure were found between these different anatomical sites within individuals, there were profound differences between CD and non-IBD controls, specifically a significant increase in the abundance of the Proteobacteria and a decrease in abundance of Firmicutes and Bacteroidetes in CD patients. Comparisons between CD and UC patients revealed a greater abundance of *Escherichia*, *Ruminococcus* (*R. gnavus*), *Clostridium*, *Cetobacterium*, *Peptostreptococcus* in CD patients, and *Faecalibacterium*, *Blautia*, *Bifidobacterium*, *Roseburia* and *Citrobacter* in UC patients [96].

Some studies compared the influence of different sampling sites and methods on the MAM composition of patients with IBD. For example, through deep sequencing of luminal brush samples, mucosal biopsies and LCM, Lavelle and colleagues demonstrated spatial differentiation of the microbial community in UC patients compared with controls [59]. A recent study confirmed that the microbiome of stool, luminal contents and biopsy were significantly different in UC patients based on 16S rRNA sequencing and, although the sample size was modest, the results suggested that luminal content aspirates obtained during colonoscopy were a better predictor of UC [97]. Collectively, these studies that indicate mucosa-associated dysbiosis in IBD patients support the notion that CD and UC may be distinguished based on their MAM structure.

### Irritable bowel syndrome

Significant differences have been reported between MAM and LM in patients suffering from IBS [98–101]. However, results are inconsistent for direct associations between IBS and LM [100,102–104] or IBS and MAM [98,101,105], which may be attributable to bowel preparation [105], the small numbers of samples or selection criteria and detection methods introducing bias [106]. In a recent study focusing on diarrhoea-predominant IBS (IBS-D) [107], MAM diversity was significantly higher than LM diversity in IBS-D patients (*n* = 69); furthermore, LM diversity in IBS-D patients was not significantly different from healthy controls (*n* = 20), while MAM diversity was significantly altered compared with healthy controls [107]. These findings correlated with the fact that a greater number of functional genes were altered in MAM compared with LM in IBS-D. Interestingly, while there was no correlation between LM composition and clinical symptoms in IBS-D patients, there was a close relationship between MAM composition and clinical symptoms reflecting MAM capacity to influence intestinal epithelial and immune cells [107].

In another recent study, 16S rRNA gene amplicon sequencing was used to compare MAM composition in samples taken using an endoscopic brush from the terminal ileum and sigmoid colon of patients with IBS (17 IBS-D patients, seven constipation-predominant IBS (IBS-C) patients and ten healthy controls) [108]. The genera *Ruminococcus*, *Akkermansia*, *Butyrivibrio*, *Methylobacterium* and *Microbacterium*, and the family *Erysipelotrichaceae* were significantly more abundant in the IBS-C group than the control group, and the abundances of *Streptococcus*, *Acidaminococcus*, *Butyricicoccus* and *Parvimonas* were significantly higher in the IBS-D group than the control group. In addition, the proportion of genes responsible for the secretion system and LPS biosynthesis were significantly higher and the proportion of genes responsible for methane metabolism, lysine biosynthesis and enzyme families were significantly lower in the IBS-D group than in the IBS-C group [108].

Together, these recent studies suggest that the structure and function of MAM is different amongst subtypes of IBS and may play a crucial role in IBS symptom generation. Furthermore, a study examining CD, UC and IBS patients showed that mucosa-associated *F. prausnitzii* and *E. coli* co-abundance can be used to distinguish IBS and IBD phenotypes [109].

### Colorectal cancer

The role of mucus and microbiota in cancer progression has been extensively reviewed (for a recent review see [110]). The structure of the microbiota in cancerous tissue differs significantly differs from that of the intestinal lumen [61] but colorectal tumours demonstrate variation in MAM composition amongst different studies.

Among the diverse set of bacterial taxa identified in these studies, *Fusobacterium*, *B. fragilis* and *Parvimonas micra* were consistently associated with tumour tissues [77]. In addition to Bacteroidetes and Firmicutes, environmental non-fermentative Proteobacteria were found in colonic crypts from CRC patients [77]. Furthermore, right-side (ascending, proximal) tumours were marked by the presence of a bacterial biofilm, unlike left-side (descending, distal) tumours [111–113]. 16S rRNA gene sequencing revealed that right and left crypt- and mucosa-associated bacterial communities were significantly different [77]. Moreover, tumour-associated microbiota varied with tumour stage and progression of the disease [114–116]. Carcinogenic subtypes *pks+ E. coli* and enterotoxigenic *B. fragilis* (ETBF) formed biofilms and the reduction in the mucus layer by ETBF allowed *pks+ E. coli* to reach the intestinal epithelium [111,117–118]. Recently, a preliminary study showed that the microbiota of intestinal lavage fluid obtained from CRC patients preparing for laparoscopic colorectal resection closely relates to MAM [119].

A recent computational study to determine a consensus mucosal microbiome for CRC analysed 924 tumours from eight independent RNA-Seq datasets; they identified a cancer-specific set of 114 microbial species that were associated with tumours and found in all of the studies evaluated (https://crc-microbiome.stanford.edu). Firmicutes, Bacteroidetes, Proteobacteria and Actinobacteria were among the four most abundant phyla in the CRC mucosal microbiome. Some species in the genus *Clostridium* were depleted while *Fusobacterium nucleatum* was one of the most enriched bacterial species in tumours [120]; the proportion of *F. nucleatum* gradually increased from rectum to caecum in *F. nucleatum*-high CRC patients [112]. The colonisation of the intestinal mucus layer by microbes such as *F. nucleatum* originally found in the oral cavity [121] was shown to further promote tumour progression in CRC patients [122] and in mice harbouring a human microbiome [123]. The interaction between pathogenic *Clostridioides difficile* and *F. nucleatum* in the intestinal mucus layer [124] is also relevant as cancer patients have an increased risk of *C. difficile* infection (CDI) compared with non-cancer patients.

While CRC is characterised by progression from adenoma to carcinoma, its development may also follow the events of IBD, termed colitis-associated colorectal cancer (CAC). A recent study investigated the bacterial and fungal composition of the MAM of patients suffering CAC, sporadic cancer (SC) and of healthy subjects using 16S MiSeq and pyrosequencing [75]. MAM in cancer patients was characterised by reduced overall microbial diversity but no change in the fungal community. Compared with SC, CAC was characterised by an increase in the abundance of the family *Enterobacteriacae* and the genus *Sphingomonas*, and a decrease in the abundance of the genera *Fusobacterium* and *Ruminococcus*. Although the cohort was limited in number (7 patients with CAC, 10 patients with SC), this study confirmed that MAM was altered in IBD and SC and provided the first evidence for the existence of an altered bacterial microbiota in CAC which differed from SC patients [75].

## Perspectives

- MAM differs from luminal and FM in composition and functionality. In CRC, IBD and IBS, MAM structure and function allow diseased and healthy individuals to be distinguished. However, the limited number of studies on MAM suffer from the small sample size, confounding effects of colonic lavage, variability in the anatomical site being sampled, the nature of the sample and the sampling and profiling method prevents robust comparative analyses.
- The development of more accurate, less invasive and standardised sampling methods is critical for future research on the relationship between MAM and human diseases (not only those requiring endoscopy); this requires collaboration between clinicians, biologists and bioinformaticians to work together to develop procedures that reduce risks/inconvenience to patients and alleviate confounding factors associated with existing methodologies.
- In the future, MAM communities require comprehensive analyses of their function through approaches such as metabolomics. In addition, since human intestinal biopsies or mucosal lavages contain host material, they could also be used to identify receptors of the interaction with MAM, but this would require optimisation of current sample preparation and procedures to enable both microbial and host analyses. Advanced models that accurately recapitulate the human mucosal interface are warranted to determine the mechanisms underpinning the relationship between MAM and human diseases.

## Abbreviations

CAC: colitis-associated colorectal cancer
CD: Crohn's disease
CDI: *C. difficile* infection
CRC: colorectal cancer
FISH: fluorescence *in situ* hybridisation
FM: faecal microbiota
GI: gastrointestinal
IBD: inflammatory bowel disease
IBS: irritable bowel syndrome
LCM: laser capture microdissection
LM: luminal microbiota
MAM: mucosa-associated microbiota
mice: mucin 2, MUC2 (human), muc2
SC: sporadic cancer
UC: ulcerative colitis
